# United States Veterinary Medical Association

**Published:** 1890-08

**Authors:** W. Horace Hoskins

**Affiliations:** Secretary


### UNITED STATES VETERINARY MEDICAL ASSOCIATION.

The prospects of a large turnout of Eastern veterinarians to the Chicago meeting in September are very promising. Already favorable responses are at hand from members in Maine, New Hampshire, Massachusetts, New York, Pennsylvania, Delaware, Maryland and the District of Columbia, and the large delegation from Philadelphia and vicinity almost assures us of the required number for a special train.

A letter just received announces the subject of Prof. Liautard's paper as that of "Veterinary Jurisprudence," and his long years of experience in the commercial centre of our country, and his association in the profession from its infancy in this country, filling a very leading position as a public teacher and educator, peculiarly fits him to deal thorougly with this important subject.

The subject of Dr. Tait Butler's paper will be announced at a later date, together with a synopsis of the committee reports in preparation.

W. Horace Hoskins, *Secretary*.

